# A Longitudinal Study of Spirituality, Character Strengths, Subjective Well-Being, and Prosociality in Middle School Adolescents

**DOI:** 10.3389/fpsyg.2019.00377

**Published:** 2019-02-27

**Authors:** Ariel Kor, Steven Pirutinsky, Mario Mikulincer, Anat Shoshani, Lisa Miller

**Affiliations:** ^1^Teachers College, Columbia University, New York, NY, United States; ^2^Baruch Ivcher School of Psychology, Interdisciplinary Center Herzliya, Herzliya, Israel; ^3^Touro College, New York, NY, United States

**Keywords:** spirituality, character strengths, well-being, adolescence, prosociality

## Abstract

Using data from 1,352 middle-school Israeli adolescents, the current study examines the interface of spirituality and character strengths and its longitudinal contribution to subjective well-being and prosociality. Participants were approached three times over a 14-months period and completed measures of character strengths, spirituality, subjective well-being (positive emotions, life satisfaction), and prosociality. Findings revealed a fourth-factor structure of character strengths that included the typical tripartite classification of intrapersonal, interpersonal, and intellectual strengths together with spirituality emerging as a statistically autonomous factor. Spirituality was stable over time and contributed to higher subjective well-being and prosociality both cross-sectionally and longitudinally. Discussion focuses on spirituality as a fundamental character strength and an important aspect of positive development.

## Introduction

In recent years, the arena of spirituality has garnered renewed interest in wide-ranging disciplines, including health (e.g., Hill and Pargament, 2003), education (e.g., Miller, 2009), clinical psychology and psychotherapy (e.g., Miller and Kelley, 2006), personality (e.g., Emmons, 1999), family studies (e.g., Tarakeshwar et al., 2001), and developmental psychology (e.g., Benson et al., 2003). Moreover, research in positive psychology and thriving consistently indicates that spirituality is associated with psychological adjustment and well-being (e.g., King and Benson, 2006; Johnson, 2008; Saroglou et al., 2008). Recently, major theoretical reviews in developmental psychology have emphasized the prominent role that spirituality plays in character formation and positive development (e.g., Benson et al., 2012a; Pargament et al., 2013; King and Boyatzis, 2015). However, while spirituality is highly relevant to youth development, the ways they relate to other character strengths and contribute to adolescents’ psychological adjustment and well-being remain understudied.

A recent review of the descriptive attempts to define spirituality shows that this field of study is overrun with confusion (e.g., King and Boyatzis, 2015), though it is slowly moving toward greater clarity. This confusion is partly attributable to the concepts of spirituality and religiousness that initially were used synonymously (James, 1982), and more recently have begun to diverge in both psychology research and popular culture (Koenig et al., 2001). Spirituality is often characterized as the degree to which people affirm and honor a transcendent or scared force in their life that often provides a profound sense of meaning and purpose (Benson et al., 2005). Thus, spirituality does not necessarily require belief in God or adherence to a particular religious denomination, whereas religiousness include affiliation to such a denomination and commitment to specific religious beliefs and practices. Despite these differences, however, spirituality and religiousness are fairly highly correlated. Treating them as completely separate constructs may not accurately reflect how spirituality and religion are experienced in the lives of people (e.g., Zinnbauer and Pargament, 2005; Crawford et al., 2006). Therefore, recent conceptualizations of spirituality view the construct as a broader term encompassing a search for the sacred both within and outside traditional religious denominations (e.g., Cragun et al., 2015), with spirituality being perceived as more individually constructed and religiousness as more socially constructed (e.g., Kapuscinski and Masters, 2010; Benson et al., 2012a). According to Koenig et al. (2001), spirituality is a personal exploration of core questions about life, meaning, and transcendent forces, which may (or may not) lead to commitment to specific religious beliefs and practices. Researchers of youth development rely on similar definitions of spirituality, such as the capacity for self-transcendence, with the goal of finding meaning, awareness, purpose and connectedness (e.g., Johnson, 2008; Lerner et al., 2008; Benson et al., 2012a).

### Spiritual Development and Well-Being in Adolescence

Although spiritual development in adolescence as a research subject has been almost absent from the developmental psychology literature (Benson et al., 2003), it is well agreed that adolescence is a sensitive period for developing spiritual belief and engagement (Good and Willoughby, 2008). The unique developmental changes that occur during adolescence, including increased capacity for abstract thought, development of metacognitive abilities, and identity formation, provide a particular opportunity for spiritual awakening (Templeton and Eccles, 2006). Stress and heightened negative emotions during adolescence are other important factors that may prompt spiritual exploration and commitment (Zinnbauer and Pargament, 1998). Research, although not providing conclusive evidence, indicate that most adolescents engage in spiritual exploration and have spiritual-like experiences and most of the spiritual commitments to beliefs and practices made during adolescence tend to persist throughout adulthood (Good and Willoughby, 2008). In the most comprehensive research on spirituality in adolescence, Benson and Scales (2009) viewed spiritual development as the dynamic interplay of three main psychological processes: (a) Being aware of the strengths, wonder, and beauty both within the self and the world in ways that cultivate meaning, identity, and purpose; (b) seeking and experiencing significance and interdependence in relationships with others or transcendent figures (God or a higher power) that provide meaning to life over time; and (c) authentically expressing one’s values, passions, and identity through activities, practices and relationships that promote a sense of inner wholeness and harmony.

In an attempt to obtain a global picture of youth spirituality, Benson et al. (2012b) surveyed 6,725 young people, between ages of 12 and 25, from eight countries in five continents. Findings yielded two main categories of youth spirituality. One category includes psychological processes that underlie spiritual development: connecting with others through pro-social beliefs and actions, discovering meaning, mindfulness, and alignment with values and action. The second category deals with religious and spiritual engagement, including spiritual practices, apprehension of God/Force, spiritual experiences, and religious practice. These findings were replicated across the eight countries and across different religious denominations. In addition, latent class analysis (LCA) rendered six types of spiritual development, which emphasized various combinations of spiritual variables (e.g., praying and experiencing a higher power or God) and religious variables (e.g., learning sacred texts and attending religious services). While this study greatly contributes to understanding youth spiritual development, its major limitation is that it was not longitudinal and therefore does not address developmental aspects.

Recent studies have also explored how spirituality is a potentially important anchor for socio-emotional adjustment. For example, spirituality has been found to shield adolescents against risky behavior, such as delinquency, substance abuse, sexual promiscuity, and emotional problems, such as depression, anxiety, and suicidality (e.g., Sinha et al., 2007; Desrosiers and Miller, 2008; King and Roeser, 2009). Studies have also demonstrated that spirituality is significantly associated with indicators of subjective well-being – higher levels of positive emotions (e.g., Ciarrocchi and Deneke, 2005; Holder et al., 2010; Smith et al., 2012) and more life satisfaction (e.g., Kelley and Miller, 2007; Kim et al., 2013). These findings are important for understanding the contribution of spirituality to socio-emotional adjustment, because recurrent experience of positive emotions, which are an important component of spiritual practices (e.g., Van Cappellen and Rimé, 2014), have been shown to build psychological, physical, and social resources that enhance one’s ability to deal with life hardships (e.g., Fredrickson et al., 2008; Cohn et al., 2009). There is also mounting evidence that adolescents’ spirituality contributes to prosociality – more compassionate feelings and behaviors toward needy others, higher levels of civic engagement, and heightened peer likeability, which, in turn, facilitate social adjustment and functioning (e.g., King and Furrow, 2004; Hardy and Carlo, 2005; Roehlkepartain et al., 2006). Some studies also point to spirituality as a source of optimism for good outcomes and of unwillingness to fall into despair during difficult times (Kloos and Moore, 2000; Park, 2005; Marques et al., 2013). Although optimism is considered a personality trait that is relatively stable over time (e.g., Cardemil et al., 2002), there is empirical evidence that optimism is reinforced by spiritual experiences (e.g., Gillham and Reivich, 2004; Gillham et al., 2007). Mofidi et al. (2007) noted that the relationship between spirituality and optimism is often bidirectional in that spirituality may promote optimism and optimism may support spirituality.

### Spirituality as a Character Strength

The relatively new focus on character strengths and virtues within the positive psychology movement helps to locate spirituality as a human character strength. Synthesizing volumes of inventories of human character strengths, both historical and contemporary, Peterson and Seligman (2004) developed the values-in-action (VIA) framework – a hierarchical classification of two main characteristics of the good character: virtues and character strengths. Virtues are conceptualized as positive traits of character that enable individuals to thrive and flourish (Park and Peterson, 2006). Based on a survey of religious, philosophical and historical texts, Peterson and Seligman (2004) identified six core human virtues: wisdom and knowledge, courage, humanity, justice, temperance, and transcendence. Character strengths are conceptualized as the psychological constituents, mechanisms or processes that define the virtues (Park, 2004). Peterson and Seligman (2004) proposed 24 character strengths that are embodied in the six core virtues. These virtues and strengths include the following: (1) Wisdom and Knowledge (curiosity, love of learning, judgment, creativity, perspective); (2) Courage (bravery, perseverance, honesty, zest); (3) Humanity (love, kindness, social intelligence); (4) Justice (teamwork, fairness, leadership); (5) Temperance (forgiveness, humility, prudence, self-regulation); and (6) Transcendence (appreciation of beauty, gratitude, hope, humor, spirituality). In this VIA framework, spirituality is considered a character strength embodied within the virtue of transcendence. That is, spirituality is not viewed as a discrete category by its own but only as part of the transcendence category that include other strengths of appreciation of beauty, gratitude, hope, and humor.

Research on Peterson and Seligman’s (2004) scheme of strength categories has relied on the values in action inventory of strengths (VIA-IS) and its adaptation for studying children and adolescents – VIA-Youth (e.g., Seligman et al., 2005; Park et al., 2006; Toner et al., 2012).

Overall, empirical evidence for the validity of the six-virtue classification is mixed, with inconsistent numbers of higher-order factors and discrepancies in their compositions (e.g., Gillham et al., 2011; Ruch et al., 2013; Weber et al., 2013; Shoshani and Slone, 2016). However, they all contain factors that represent interpersonal, intrapersonal, and intellectual strengths, with an additional factor of transcendence that includes the strength of spirituality.

The VIA-IS for youth has been validated and found to be related to socio-emotional adjustment and psychological functioning (Park, 2004; Shoshani and Slone, 2013). Specifically, studies with elementary and middle school students have indicated that interpersonal strengths are related to better social functioning, wisdom strengths to more academic achievements, and temperance and transcendence strengths to higher levels of positive emotions and life satisfaction and fewer behavioral and emotional problems (Gillham et al., 2011; Shoshani and Aviv, 2012; Shoshani and Slone, 2013, 2016).

One basic issue that our study attempts to elucidate concerns the uniqueness of spirituality as a character strength. In other words, it aims to explore whether spirituality is only part of the broader category of transcendence or it is a discrete high-order category of strengths in its own. Although spirituality has been suggested to be only a component of the transcendence category, Peterson and Seligman (2004) themselves raised doubts about the composition of the transcendence factor and postulated that they “would not be surprised if this final grouping is revised – collapsed or combined…in subsequent editions” (p. 519). Moreover, Piedmont (1999) viewed spirituality as a basic organizing principle of human personality that shapes people’s life. He argued that spirituality represents a hierarchically structured domain of psychological functioning that directs, drives, and selects behaviors in both secular and religious contexts. In support of this view, Piedmont (1999) provided convincing evidence that spirituality represents a unique personality domain that does not overlap with other high-order personality traits.

Spirituality may also shape the ways other character strengths and virtues operate. Spirituality can add more passion and meaning to people’s intrapersonal and intellectual aspirations; it can moderate how people interact with others; it can redefine the goals people pursue; and it can help people in reappraising life events and transcending hardships and difficulties (e.g., Piedmont et al., 2009; Rican and Janosova, 2010). Thus, spirituality may be a distinctive psychological domain of comparable breadth to the virtues contained in the VIA classification and ought to be considered a potential distinct major category of character.

In the current study, we follow Piedmont’s (1999) claim that spirituality may be an independent dimension of personality or character altogether, and argue that the findings reported using the VIA classification may result from a narrow and incomplete operationalization of spirituality. In the VIA inventory of strengths, spirituality is operationalized as a belief in and commitment to the transcendent (non-material) aspects of life (Peterson and Seligman, 2004, p. 519). However, this operationalization fails to capture the complex, multidimensional nature of spirituality (e.g., King and Boyatzis, 2015).

### The Current Study

The primary aim of the current longitudinal study is to examine the relationship between spirituality, character strengths, subjective well-being (positive emotions, life satisfaction), and prosociality throughout middle school adolescence. As reviewed above, previous research has established that spirituality is an important character strength and a correlate of both subjective well-being and prosociality. However, the lack of longitudinal research hampers causal and directional conclusions (King and Boyatzis, 2015). It is, therefore, important to note that the direction of causality in the field of spiritual development remains murky at best, which emphasizes the need for research designs that are longitudinal and that test the contribution of spirituality to socio-emotional adjustment and functioning over time (King and Boyatzis, 2015).

We expect to find that spirituality is a discrete, high-order factor of character, and that the current VIA definition of spirituality as part of “transcendence,” which includes other character strengths such as gratitude, humor, and hope, is lacking. Moreover, previous studies have found that spirituality is stable during adolescent development (Good et al., 2011; Lopez et al., 2011; Pearce and Denton, 2011), and we expect to further demonstrate this. We also predict that spirituality would longitudinally contribute to positive emotions, life satisfaction, and prosociality during this period.

## Materials and Methods

### Participants

The sample included 1,352 Israeli adolescents, 655 (48%) girls and 696 (51%) boys, ranging in age from 13 to 17 (*M* = 13.43, *SD* = 0.98). They were recruited from eight middle schools across Israel and were in grades 7–9. Participants were assessed at three time points in a period of 14 months, with 98% of them (*n* = 1,328) completing all three waves. The vast majority of the participants were Jewish (85%) although a minority (15%) identified as Christian and Muslim.

### Procedure

Ethical approval for the study was obtained from the Chief Scientist of the Ministry of Education in Israel as well as from the Institutional Review Board (IRB) of IDC Herzliya and Teachers College, Columbia University. Authorization for running the study was also obtained from each of the school principals, and written consent was obtained from each participant and their parents. Participants were guaranteed confidentiality and were assured that they could withdraw from the study at any point, without having to provide a reason for doing so. The first wave of data collection (Time 1) took place at the beginning of the 2015 academic year (September). The second wave of measurement (Time 2) took place at the end of the academic year (June 2016) and the third wave (Time 3) occurred in November 2016. We will continue to collect data over the coming years. The same set of scales were completed at the three time points.

### Measures

Character strengths were assessed with a Hebrew version of the VIA Inventory of Strengths for Youth – Short Form (VIA-Y; Park and Peterson, 2005, 2006). This scale includes 96 items and was designed for children and adolescents between the ages of 10 and 17 years. The VIA-Y assesses 24 character strengths (4 items per strength): Curiosity, love of learning, judgment, creativity, perspective, bravery, perseverance, honesty, zest, love, kindness, social intelligence, teamwork, fairness, leadership, forgiveness, humility, prudence, self-regulation, appreciation of beauty, gratitude, hope, humor, and spirituality. The VIA-Y short form had good psychometric qualities, with alpha scores ranging from 0.84 to 0.87 and has been already used among Israeli children and adolescents (e.g., Shoshani and Aviv, 2012; Shoshani and Slone, 2013). Based on the VIA’s institute coding schema, we computed 24 total scores for each participant representing each of the strengths assessed in the questionnaire.

Participants also completed the Life Orientation Test-Revised (LOT-R: Scheier et al., 1994) in order to assess optimism as a character strength that is not in included in the VIA-Y. The LOT-R is a 10-item questionnaire tapping adolescents’ optimism and their positive expectations for the future. This measure includes positively phrased items reflecting optimism (e.g., “I’m always hopeful about my future”), and negatively phrased items that reflect pessimism (e.g., “Things usually go wrong for me”). Participants rated their agreement with each item on a 5-point scale, ranging from 1 (*strongly disagree*) to 5 (*strongly agree*). The LOT-R has been used in hundreds of studies and has been consistently found to be a reliable and valid scale (e.g., Steca et al., 2015). In the current study, Cronbach Alphas were acceptable (from 0.70 to 0.71) at the three time points. Then, we computed a total optimism score for each participant at each time point by averaging the 10 items.

We assessed spirituality with four different self-report scales. First, participants completed the Faith Maturity Scale (FMS, Benson et al., 1993). The FMS consists of 12 items that assess the extent to which spirituality plays a role in a person’s life. It includes items tapping the extent to which spirituality influences one’s inclination to help others (e.g., “I feel a deep sense of responsibility for reducing pain and suffering in the world”) and one’s closeness to God (e.g., “Every day I see evidence that God or a higher force is active in the world”). Items were rated on a 5-point scale, ranging from 1 (*never true*) to 5 (*always true*). Past studies have found this scale to be reliable and valid (e.g., Benson et al., 1993; Hall et al., 2016). In the current study, Cronbach Alphas were acceptable (from 0.93 to 0.95) at the three time points. On this basis, we computed a total score for each participant at each time point by averaging the items.

Second, participants completed the Duke University Religious Index (DUREL; Koenig et al., 1997). The DUREL consists of two items tapping participation in organized and non-organized religion practices, (e.g., “How often do you attend synagogue or other religious meetings?”, “How often do you spend time in private religious activities, such as prayer, meditation or Bible study?”) and three items tapping intrinsic religiosity (e.g., “My religious beliefs are what really lie behind my whole approach to life”). Items related to frequency of practice are scored on a 6-point scale, ranging from 1 (*rarely or never*) to 6 (*more than once a day*). The remaining items are scored on a 5-point scale, ranging from 1 (*definitely not true*) to 5 (*definitely true of me*). Previous studies have provided evidence on the reliability and validity of this scale (e.g., Koenig et al., 1997; Freire de Medeiros et al., 2017). In the current study, we computed two total scores for each participant at each time point – participation in religious practices (αs ranging from 0.72 to 0.76) and intrinsic religiosity (αs ranging from 0.90 to 0.92), by averaging the relevant items.

Third, participants completed three items from the Personal Devotion scale (PDS, Kendler et al., 1997): “How important are religious or spiritual beliefs in your daily life?”; “When you have problems in your life, how often do you seek spiritual comfort?”; and “When you have decisions to make in your daily life, how often do you ask yourself what God would want you to do?” Items were rated on a 5-point scale, either from 1 (*never important*) to 5 (*very* important), or from 1 (never) to 5 (very often). On this basis, we computed a total score for each participant at each time point by averaging the items. Cronbach Alphas were acceptable for the three PDS items (αs ranging from 0.80 to 0.82).

Fourth, participants completed four items from the Spiritual Transcendence Scale (STS: Piedmont, 1999), tapping the ability to view life from a more objective perspective, to perceive the fundamental unity in the world, and to see a larger meaning in human existence. In the current study, Cronbach Alphas were acceptable for the four STS items (αs ranging from 0.75 to 0.87). We computed total scores for each participant at each time point by averaging items in the scale.

Subjective well-being was assessed with two scales tapping the two indicators of this construct: positive emotions and life satisfaction. Positive emotions were assessed with the positive affect subscale of the Positive and Negative Affect Schedule for Children (PANAS-C; Ebesutani et al., 2012). Using a 5-point scale, ranging from 1 (*very slightly*) to 5 (*very much*), participants rated the extent to which five adjectives representing positive emotions describe themselves over the last few weeks. The PANAS-C has been shown to have high reliability and validity (e.g., Ebesutani et al., 2012; Shoshani and Shwartz, 2018). For the present study, we only rely on these five positive emotions items, since we mainly focus on the link between spirituality and subjective well-being (Smith et al., 2012). The Cronbach alphas for this subscale were acceptable (from 0.79 to 0.82) at the three time points. On this basis, we computed a total score for each participant at each time point by adding the five items. Life satisfaction was assessed with the Satisfaction with Life Scale (SWLS; Diener et al., 1985). There are five items in the scale (e.g., “In most ways, my life is close to my ideal”), which are rated on a 7-point scale, ranging from 1 (*strongly disagree*) to 7 (*strongly agree*). The total score is a sum of a participant’s responses. Previous studies have found the SWLS to have high reliability and validity (e.g., Pavot and Diener, 2008). In the current study, Cronbach Alphas were acceptable (from 0.85 to 0.87) at the three time points.

Prosociality was assessed with the prosociality subscale of the Strengths and Difficulties Questionnaire (SDQ; Goodman et al., 1998). This subscale includes five items (e.g., “I share readily with other children, for example toys, treats, pencils”) and participants rated how much each items described them best, using a 3-point scale, ranging from 1 (*not true*) to 3 (*certainly true*). Previous studies have provided evidence on the reliability and validity of this subscale (e.g., Goodman et al., 1998; Mansbach-Kleinfeld et al., 2010; Shoshani et al., 2016; Shoshani and Russo-Netzer, 2017). In the current study, Cronbach Alphas were acceptable (from 0.69 to 0.74) at the three time points. On this basis, we computed a total score for each participant at each time point by summing up the five items.

## Results

### Is Spirituality a Unique Aspect of Youth Character Strengths?

In order to assess our hypothesis that spirituality is a discrete aspect of youth character strengths, we conducted an exploratory factor analysis (principal axis factoring) on the 24 VIA strengths, the optimism score, and the five spirituality scores from Time 1. Data was appropriate for exploratory factor analysis (Kaiser-Meyer-Olkin = 0.95; Bartlett’s test of sphericity: χ^2^ (435) = 29520, *p* < 0.001). Results indicated that five factors had eigenvalues greater than 1 (ranging from 10.53 to 1.18). However, examination of the scree plot and a parallel analysis using a bootstrapping method (O’Connor, 2000) suggested that a four-factor solution accounting for 57.28% of the variance fit the data optimally. Therefore, we retained the four-factor solution and rotated these factors using non-orthogonal, direct oblimin rotation. Six items loaded lower than 0.40 on each of the rotated factors or strongly cross-loaded on multiple factors (VIA scores of bravery, gratitude, perseverance, kindness, and social intelligence, and the STS score). These items were thus dropped and the remaining 24 items were reanalyzed. This yielded a clear four-factor solution with eigenvalues ranging from 7.94 to 1.31, and accounting for 59.04% of variance. All loadings were higher than 0.40 and no cross-loading was observed (see Table 1).

**Table 1:** Pattern matrix after rotation for the final four-factor solution.

	Interpersonal		Intrapersonal	Intellectual
	strengths	Spirituality	strengths	strengths
Prudence	0.72			
Fairness	0.72			
Judgment	0.69			
Self-regulation	0.64			
Forgiveness	0.61			
Humility	0.58			
Honesty	0.58			
Teamwork	0.57			
Personal devotion		0.91		
Intrinsic religiosity		0.89		
Faith maturity		0.87		
VIA spirituality		0.82		
Religious practice		0.73		
Zest			0.74	
Love			0.73	
Leadership			0.72	
Optimism			0.66	
Humor			0.65	
Hope			0.60	
Perspective			0.60	
Curiosity				0.81
Creativity				0.74
Appreciation of				0.62
beauty
Love of learning				0.61


*Loadings less than 0.40 are omitted.*

As reported Table 1, this analysis yielded four factors labeled Interpersonal strengths, spirituality, intrapersonal strengths, and intellectual strengths. The factors representing interpersonal, intrapersonal, and intellectual strengths included all VIA scores retained, with the exception of the VIA spirituality score. The optimism LOT-R score loaded on the intrapersonal strengths factor, and the spirituality factor included the VIA spirituality score and scores on intrinsic religiosity, personal devotion, religious practice, and faith maturity scales. Factor correlations indicated that the spirituality factor was moderately associated with the intrapersonal strengths factor (0.26), weakly with the interpersonal strengths factor (0.14), and minimally with the intellectual strengths factor (0.08). The other three strengths factors showed stronger correlations between them (ranging from 0.36 to 0.42). The emergence of a spirituality factor incorporating the various aspects of spirituality and separated from other character strengths supported the hypothesis that spirituality is a unique, although related, aspect of youth character strengths.

To confirm the four-factor structure described above, we then conducted a series of multi-group confirmatory factor analyses (CFA) that tested configural invariances across boys and girls. Fit for CFA models was assessed using the guidelines suggested by Kenny (2015), and included RMSEA less than or equal to 0.08, CFI approaching 0.90, and decreasing BIC. Given the large sample size, non-significant chi-square values were interpreted cautiously. Results indicated that the above described four-factor model displayed reasonable fit among boys [χ^2^(246) = 895, *p* < 0.001, CFI = 0.90, RMSEA = 0.07] and among girls [χ^2^(246) = 995, *p* < 0.001, CFI = 0.88, RMSEA = 0.08]. The model for the entire sample was also adequate [χ^2^(246) = 1597, *p* < 0.001, CFI = 0.89, RMSEA = 0.07], suggesting that the four-factor structure described in Table 1 was configurally invariant across genders.^1^

To further explore the associations of the spirituality measures and the other character strengths, we conducted a LCA to select the best fitting categorization of participants. This analysis built upon the factor analytic model described above and added a fifth latent categorical variable that predicted means levels on each of the four factors. A series of models with the number of groups varying from 3 to 6 were run. Following Nylund et al. (2007), model fit was assessed by lower BIC, reasonable class size relative to sample size (minimum 20), entropy approaching 0.80, and non-significant VLMR, LRT, and bootstrapping likelihood ratio tests. Although inferential log-likelihood tests suggested that a five-latent class model fit the data best, this model had higher BIC, lower membership probabilities, and extremely small class sizes, suggesting that it was likely over-parameterized. A model with four latent classes provided the best balance between fit and parsimony, as indicated by BIC, entropy, membership probabilities, and class sizes (see Table 2).

**Table 2:** Model fit and summary statistics for LCA models classifying participants according to their scores in the spirituality and the other three character strengths factors.

Number of		Class		Membership
classes	BIC	sizes	Entropy	probabilities	VLMR	LRT	Bootstrap
3	11674	566–30	0.71	0.57-0.88	38.78	37.50	38.58^∗∗^
4	11666	471–30	0.75	0.74-0.89	42.73^∗^	41.53^∗^	42.73^∗∗^
5	11700	560–2	0.80	0.60-0.88	25.31^∗^	24.60^∗^	25.31^∗∗^
6	11705	468–3	0.80	0.60-0.90	4.48	4.35	4.48


*Vuong-Lo-Mendell-Rubin (VLMR), Lo-Mendell-Rubin Adjusted (LRT), and parametric bootstrapped (Bootstrap) likelihood ratio tests inferentially compared a model with k classes to a model with k-1 classes. ^∗^*p* < 0.05; ^∗∗^*p*< 0.001.*

**FIGURE 1:**
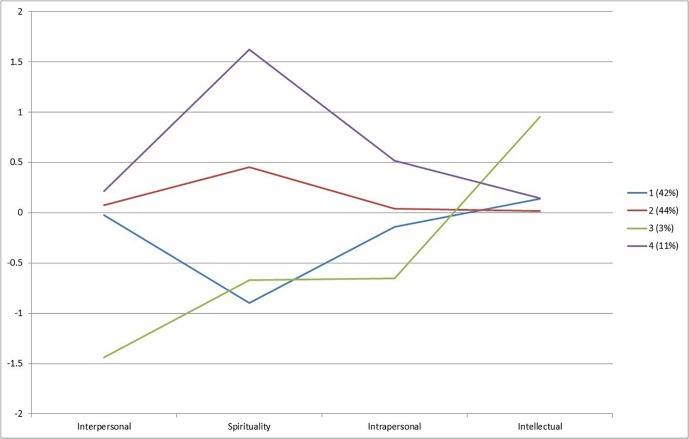
Differences between four latent classes on mean levels of each underlying continuous latent factor of positive character at Time 1. Scores represent standardized *Z* scores on the latent variable representing each facet of character.

Parameters for this model indicate that the four groups can be characterized according to the following participants’ scores at Time 1 (see Figure 1): Low spirituality and average scores in the other three strengths factors (Class 1); medium spirituality and high-average scores in the other three strengths factors (Class 2); low spirituality, low interpersonal strengths, and high intellectual strengths scores (Class 3); and high spirituality and high intrapersonal strengths scores (Class 4). The vast majority of participants were classified in Class 1 (42%), and Class 2 (44%) and participants in other latent classes were relatively rare (3 and 11%). Of note, LCA analysis did not identify a latent class with participants scoring high on spirituality and low on the other three strengths factors, suggesting that most of the spiritually involved participants also scored high on other character strengths. In fact, the latent class with the highest level of spirituality included participants who scored high on the intrapersonal strengths factor.^2^

### Is Spirituality Stable Over Time?

To examine the extent to which spirituality in adolescence is stable over time, we first assessed the structural stability of the four-factor structure described in Table 1 by subjecting all variables to a CFA at Time 2 (9 months later) and Time 3 (14 months later). Results indicated that the four-factor structure of spirituality and three other character strengths continued to display reasonable fit at Time 2 [χ^2^(246) = 20002, *p* < 0.001, CFI = 0.87, RMSEA = 0.08] and Time 3 [χ^2^(246) = 908.83, *p* < 0.001, CFI = 0.89, RMSEA = 0.08].

In order to examine within-participant stability in spirituality over time, we estimated a full SEM model with the five spirituality variables that loaded high on the spirituality factor. In this model, all spirituality variables at each time point are assumed to load on a latent variable of spirituality, and stability was assessed through the standardized coefficient predicting latent spirituality at Time 2 from latent spirituality at Time 1, and latent spirituality at Time 3 from latent spirituality at Time 2. Overall, this model fit the data well [χ^2^(88) = 1195, *p* < 0.001, CFI = 0.88, RMSEA = 0.13, SRMR = 0.06]. Temporal stability of spirituality was strong [Time 2: *B* = 0.95, *SE* = 0.04, *Z* = 25.369, *p* < 0.001; Time 3: *B* = 0.97, *SE* = 0.04, *Z* = 27.96, *p* < 0.001], with 89% of the variance in latent spirituality at Time 2 explained by spirituality at Time 1, and 90% of the variance at Time 3 explained by Time 2.

We also compared stability estimates for the other three character strengths factors. Spirituality was slightly more stable than interpersonal strengths (Time 2: *B* = 0.79, *SE* = 0.05, *Z* = 16.89, *p* < 0.001; Time 3: *B* = 0.74, *SE* = 0.05, *Z* = 16.23, *p* < 0.001), intrapersonal strengths (Time 2: *B* = 0.89, *SE* = 0.07, *Z* = 13.01, *p* < 0.001; Time 3: *B* = 0.82, *SE* = 0.06, *Z* = 13.98, *p* < 0.001), and intellectual strengths (Time 2: *B* = 0.81, *SE* = 0.05, *Z* = 16.02, *p* < 0.001; Time 3: *B* = 0.82, *SE* = 0.05, *Z* = 17.74, *p* < 0.001). That is, spirituality in adolescence was largely stable over a 1-year period, and perhaps even more stable than other character strengths.

### Cross-Sectional Associations Between Spirituality, Subjective Well-Being, and Prosociality

Pearson correlations were computed in order to assess cross-sectional associations between spirituality, on the one hand, and positive emotions, life satisfaction, and prosocial behavior, on the another, at each wave of measurement. Regression weights derived from the factor analysis described above were used to calculate a participant’s score on the spirituality factor at each wave of measurement. Although there were some differences between the three time points, spirituality was found to correlate significantly but moderately with heightened life satisfaction (0.23, 0.24, and 0.16, all *p*s < 0.01), positive emotions (0.21 and 0.23, 0.14, all *p*s < 0.01), and prosociality (0.11, 0.12, 0.18, all *p*s < 0.01). We also computed multiple regressions examining the unique contribution of spirituality beyond and above the contribution of the other three high-order character strengths. These analyses indicated that spirituality made a significant unique contribution to prosociality and life satisfaction at each of the three points (β ranging from 0.10 to 0.21, all *p*s < 0.01). However, its significant association with positive emotions was no longer significant after statistically controlling for the other three character strengths (β ranging from 0.01 to 0.05). In this case, the intrapersonal strength factor was the single predictor with a significant unique contribution to positive emotions (β ranging from 0.56 to 0.62, all *p*s < 0.01). In addition, the four latent categories identified using LCA were compared on mean levels on all positive outcome variables, using ANOVA and post-hoc Scheffe tests. Results of this analysis indicated that the high spirituality/high intrapersonal character (Class 4) group generally reported significantly higher levels of positive outcomes, such as life satisfaction, prosociality, and positive emotion, as compared to other groups (see Table 3).

**Table 3:** Means, SDs, and ANOVas statistics of subjective well-being and prosociality at Time 1 according to groups derived from LCA performed on spiritual, intrapersonal, interpersonal, and intellectual character strengths at Time 1.

	Class 1		Class 2		Class 3		Class 4		
					
	*M*	*SD*	*M*	*SD*	*M*	*SD*	*M*	*SD*	*F*
Life satisfaction	23.57^ab^	6.91	25.37^ab^	6.74	17.78^a^	6.73	27.65^b^	5.26	22.49^∗∗∗^
Positive emotions	17.77^ab^	4.04	18.82^ab^	3.92	16.73^a^	4.85	20.10^b^	3.57	13.99^∗∗∗^
Prosociality	7.21^ab^	2.02	7.56^ab^	1.94	6.33^a^	2.02	8.13^b^	1.91	10.49^∗∗∗^


*^∗∗∗^*p* < 0.001; Means with similar superscripts did not differ significantly (*p* > 0.01).*

### Do Changes in Spirituality Over Time Are Associated With Subjective Well-Being and Prosociality?

To explore the temporal pattern of the relationships of spirituality with subjective well-being and prosociality, we modeled the changes in spirituality across the three waves using a latent growth mixture model. This analysis built upon the factor analytic model of spirituality described above, and modeled individual growth trajectories (intercept and slope) on the latent spirituality factor. For each participant, a latent individualized intercept and slope that best describe their baseline and rate of change were estimated. These individual growth trajectories were regressed on a categorical latent factor representing particular class memberships (LCA analysis). Then, an optimal mean slope and intercept for each class was estimated using maximum likelihood estimation. Model fit was assessed with the same LCA criteria described in the previous section.

Results indicated that a four class latent model fit the data best, as evidenced by low BIC, high membership probabilities, reasonable class sizes, and high entropy. This was confirmed by inferential log-likelihood tests, including bootstrapping (see Table 4). Examination of the mean intercept and slope parameters for these groups suggested that they can be characterized as high and increasing spirituality (Class 1, *I* = 1.55, *S* = 0.16, 11% of participants), high-average and stable spirituality (Class 2, *I* = 0.68, *S* = -0.04, 29%), low-average and stable spirituality (Class 3, *I* = -0.26, *S* = 0.01, 29%), and low and stable spirituality (Class 4, *I* = -1.12, *S* = 0.04, 31%).

**Table 4:** Model fit and summary statistics for LGMM models assigning group membership on the basis of individual spirituality growth curves.

Number of		Class		Membership
classes	BIC	Sizes	Entropy	probabilities	VLMR	LRT	Bootstrap
2	3188	279–182	0.87	0.96–0.97	18.05^∗∗^	727^∗∗^	766^∗∗^
3	2913	4224–63	0.88	0.94–0.96	11.63^∗∗^	278^∗∗^	293^∗∗^
4	2751	150–47	0.87	0.90–0.96	17.67^∗^	171^∗^	181^∗∗^
5	2735	143–4	0.89	0.91–0.95	12.75	33	35^t^


*Vuong-Lo-Mendell-Rubin (VLMR), Lo-Mendell-Rubin Adjusted (LRT), and parametric bootstrapped (Bootstrap) likelihood ratio tests inferentially compared a model with k classes to a model with k-1 classes. ^∗^*p* < 0.05; ^∗∗^*p*< 0.001. ^*t*^Bootstrapped draws failed to replicate.*

We then conducted ANOVAs comparing these groups on positive emotions, life satisfaction, and prosociality at each time point. As can be seen in Table 5, participants in Class 1 (high and increasing spirituality) reported higher life satisfaction and higher positive emotions at the three times point and higher prosociality at Times 2 and 3 than participants in Class 4 (low and stable spirituality). The high-average and low-average spirituality groups fell somewhere in between. Thus it appears that spirituality is longitudinally related to life satisfaction, positive emotions, and prosociality, and that participants with spiritual growth reported the highest levels of these variables.

**Table 5:** Means, SDs, and ANOVAs statistics of subjective well-being and prosociality at the three waves of measurement according to groups derived from individual spirituality growth curves.

	Class 1		Class 2		Class 3		Class 4		
					
	*M*	*SD*	*M*	*SD*	*M*	*SD*	*M*	*SD*	*F*
**Time 1**									
Life satisfaction	27.96^a^	5.54	24.30^ab^	6.77	24.50^ab^	6.17	23.14^bc^	6.53	6.31^∗∗∗^
Positive emotions	20.13^a^	3.52	18.22^ab^	3.80	18.25^ab^	4.09	17.50^bc^	3.98	5.04^∗∗∗^
Prosociality	7.98^a^	1.76	7.70^a^	1.89	7.47^a^	1.84	7.36^a^	1.86	1.71
**Time 2**									
Life satisfaction	27.75^a^	5.70	24.28^ab^	6.10	24.61^ab^	5.34	21.95^bc^	6.71	11.30^∗∗∗^
Positive emotions	20.11^a^	3.59	18.66^ab^	3.91	18.27^ab^	3.88	16.75^bc^	4.99	8.72^∗∗∗^
Prosociality	8.01^a^	2.01	7.73^ab^	1.78	7.60^ab^	1.91	7.14^bc^	1.86	3.54*
**Time 3**									
Life satisfaction	27.27^a^	6.65	24.52^ab^	5.81	24.41^ab^	5.92	22.53^bc^	6.04	7.54^∗∗∗^
Positive emotions	19.38^a^	4.17	18.11^ab^	4.18	17.72^ab^	4.03	17.16^bc^	4.71	3.25^∗^
Prosociality	8.08^a^	2.34	7.56^ab^	1.68	7.37^ab^	2.02	6.94^bc^	2.06	4.56^∗∗∗^


*^∗^*p* < 0.05; ^∗∗∗^*p* < 0.01; Means with similar superscripts did not differ significantly (*p* > 0.01).*

## Discussion

For over a decade now, scientific inquiry into the domain of spirituality and spiritual development has blossomed. While fraught with conceptual confusion, spirituality has been conceptualized as a core character strength contributing to flourishing and thrive (e.g., Peterson and Seligman, 2004) and spiritual development as an essential aspect of positive human development (e.g., King and Boyatzis, 2015). The current findings clearly showed that spirituality is a specific, distinct area of character strength that longitudinally contributes to positive development during adolescence. Specifically, a latent factor of spirituality was found to represent a unique category of strengths that was empirically separated from other related character strengths and to be longitudinally related to subjective well-being (positive emotions, life satisfaction) and prosociality during the 1-year study period.

The current findings concerning the structural relationship of measures of spirituality and character strengths were in line with Piedmont’s (1999) claim that spirituality is an independent dimension of character strengths. Specifically, Israeli adolescents were found to vary along a latent factor of spirituality (including measures of personal devotion, faith maturity, intrinsic religiosity, commitment to religious practices, and VIA-spirituality), which was empirically separated from other three categories of character strengths – interpersonal, intrapersonal, and intellectual. That is, exploratory and CFA yielded a four-factor structure of character strengths in which spirituality represented one of the four foundational categories of strengths. This four-factor structure does not fit the VIA’s six virtue categories, into which the 24 strengths are organized. In fact, our findings are quite similar to those of previous adolescent studies of character strengths, which implemented various adapted VIA strength scales (e.g., Toner et al., 2012; Park et al., 2017). Nonetheless, these studies included spiritual characteristics (e.g., “theological strengths” and “transcendence and vitality factors”) that were more narrowly operationalized than in our study. Peterson and Seligman (2004) claim that their conceptualization of transcendence as a high-order category of character strengths “seems mixed” (p. 519). They argue that the prototype of this category is spirituality, and the other strengths in this category (appreciation of beauty, gratitude, hope, humor, and spirituality) are expressions of a fundamental belief that there is something greater than one’s self. Our analyses clearly indicated that measures of spirituality (including the VIA spirituality strength) loaded onto a high-order category distinct from other VIA’s strengths, whereas the other strengths included in the transcendence category collapsed into other strengths categories (interpersonal, intrapersonal, and intellectual). While related to, there is no evidence suggesting that the strengths of appreciation of beauty, gratitude, hope, and humor are part of a distinct underlying component and foundation of character, as opposed to spirituality that represents a distinct category of strengths.

The emergence of a statistically autonomous spirituality factor incorporating the various aspects of spirituality measured in the current study suggests that spirituality is a distinct aspect of youth character. The implications of this finding to developmental and educational policy should not be underestimated, and educators, parents, and policy makers may need to consider incorporating spirituality into informal and formal education. Further research should more carefully explore the specific content of spirituality in adolescence, to help elucidate the mechanisms of spirituality as a foundation of character, develop theoretical models and intervention strategies, and address the challenges of integrating these concerns into the everyday lives of children and adolescents.

Adolescence has been theorized to be a time of spiritual turmoil by some (e.g., Pearce and Denton, 2011), and of spiritual stability and importance by others (e.g., Templeton and Eccles, 2006; Good et al., 2011), although there is virtually no empirical research to support either argument. Yet, Good et al. (2011), in a rare longitudinal study, found evidence for intraindividual stability in spirituality among 17–18 years-old adolescents over two time points. The current findings replicated and extended Good et al.’s (2011) findings, showing that the latent factor of spirituality we found in our sample of Israeli middle school adolescents remained stable over the three waves of measurement. Moreover, the results indicated that spirituality is slightly more stable than interpersonal, intrapersonal, and intellectual strengths. That is, it appears that spirituality among youth is largely stable over time, as shown in previous studies (Good et al., 2011; Lopez et al., 2011; Pearce and Denton, 2011).

Findings from the LCA indicated that adolescents with relatively high spirituality and high intrapersonal strengths had the highest scores on measures of subjective well-being and prosociality, whereas adolescents characterized by relatively low spirituality, high intellectual strengths, and low interpersonal strengths revealed the poorest level on these measures. This finding lend support to the hypothesis that intellectual strengths are not necessarily related to well-being during adolescence, while strengths of the heart are (e.g., Toner et al., 2012; Shoshani and Slone, 2013). Moreover, fitting previous findings (e.g., Benson et al., 2005; Ciarrocchi and Deneke, 2005; Marques et al., 2013), it seems that high levels of spirituality in adolescence tend to be closely associated with high levels of intrapersonal strengths (e.g., zest, life orientation, humor, hope, perspective) and that a mixture of them are related to heightened well-being and prosociality. Nonetheless, a thorough understanding of the mechanism by which spirituality impacts adolescent well-being is lacking. It may be that spirituality nurtures well-being and prosociality in adolescents by furnishing them with heightened purpose and connection to themselves and the divine, and with comfort during upset and disappointment (Gillham et al., 2011). Together, these findings suggest that not only is spirituality a core human mechanism, but it can also lend adolescents a life of well-being and social impact.

Findings also indicated that changes in spirituality during the 1-year study period also contributed to explain individual variations in subjective well-being and prosociality at the three waves of measurement. Adolescents with high and increasing spirituality reported the highest life satisfaction, positive emotions, and prosocial behaviors across the three time points, whereas adolescents with low and stable spirituality reported the lowest levels of these measures. These findings suggest that spirituality is longitudinally related to subjective well-being and prosociality, that adolescents who exhibit spiritual growth report the highest levels of these variables, and that positive interventions should especially target adolescents with low levels of spirituality.

The current study is one of few to focus on the interplay of spirituality, character strengths, subjective well-being, and prosociality longitudinally in a national representative sample of adolescents. Nonetheless, this project has its limitations. First of all, the study exclusively relied on self-reports. Future studies could include reports from others, such as from parents or teachers. In addition, the generalizability of the findings is compromised by the fact that the participants are all adolescents raised in Israel, a complex country with a unique set of challenges, chief among which, perhaps, is the deep social divide between the secular and religious populations (e.g., Mayseless and Salomon, 2003). As such, it is an atypical sample that, due to its unique social make-up, may be inherently averse to spirituality, which, in turn, enhances the strength of the findings, given that the sample is largely made up of secular Israelis. Future research should examine the interplay of spirituality, character strength, well-being, and prosociality across different backgrounds and cultures.

More complex longitudinal designs that track intraindividual changes from adolescence to adulthood could strengthen the validity of our longitudinal findings and examine more in-depth the long-term contributions of youth spiritual development to subjective well-being and prosocial behavior at different life domains in adulthood. In addition, future studies could examine more systematically whether and how specific dimensions of spirituality (e.g., awareness, connectedness, meaning, awe) are differentially related to specific domains of socio-emotional adjustment. Moreover, these relationships may be highly influenced by culture (e.g., Pirutinsky et al., 2011) and gender (e.g., Desrosiers and Miller, 2007), and future research would benefit from cross-cultural samples and gender comparisons.

The findings have also important implications for the planning and implementation of character education programs that aim to impact children and adolescents’ emotional, moral, and intellectual development. Spirituality has largely been absent from the discourse on character education and remains an elusive concept in the eyes of most developmental scientists. However, our findings suggest that any character education program that is devoid of spirituality may be lacking in that it ignores a foundational facet of character. Further research is needed to create a conceptual framework that will facilitate the incorporation of spirituality into character education curricula and discourse. As the popularity of character research and interventions continues to grow, additional inquiries into spirituality should deepen and become incorporated into mainstream developmental and educational sciences.

## Author Contributions

AK, SP, MM, AS, and LM have substantial contributions to the conception of the study, the acquisition, analysis, and interpretation of the research data; and preparing the manuscript for publication.

## Conflict of Interest Statement

The authors declare that the research was conducted in the absence of any commercial or financial relationships that could be construed as a potential conflict of interest.

## Footnotes

1 Measurement invariance between boys and girls was also tested by comparing a nested series of models that constrained additional parameters to be equal across groups. Results indicated that a model restraining loadings to be equal across groups but allowing intercepts to vary fit the data best as indicated by lower BIC and RMSEA values, and higher CFI. To assess if these differences reflect significant differences on the underlying factors, we compared two additional CFA models, one that fixed the intercept for each latent factor to be equal across groups, and one that allowed them to vary. Results indicated that the differences observed between genders were minimal and likely reflected over-fitting [Δχ^2^(4) = 15.74, *p* = 0.003, ΔCFI = -0.001, ΔRMSEA = 0.001, ΔAIC = 8, ΔBIC = -13].

2 A conditional LCA model that included age and gender as covariates indicated that this model displayed poorer fit to unconditional models (BIC = 19566, entropy = 0.78, class sizes: 2–478) and that neither age (Bs = -0.35 through 0.05, *p*s = 0.75 through 0.98) nor gender (Bs = -0.87 through 0.13, *p*s = 0.40 through 0.98) significantly predicted participants’ classification into latent classes.
